# Three Dimensional Printing as a Tool For Anatomical Training in Lung Surgery

**DOI:** 10.1007/s40670-023-01807-x

**Published:** 2023-06-09

**Authors:** Armelle J. A. Meershoek, Tom G. J. Loonen, Thomas J. J. Maal, Edo J. Hekma, Niek Hugen

**Affiliations:** 1 Department of Surgery, Rijnstate, Arnhem, the Netherlands; 2 3D Lab, Radboud University Medical Center, Nijmegen, The Netherlands; 3 Department of Surgery, Radboud University Medical Center, Nijmegen, The Netherlands; 4 Department of Surgery, Netherlands Cancer Institute, Amsterdam, The Netherlands

**Keywords:** Thoracic surgery, Training, 3D model, Anatomy

## Abstract

**Objective:**

Pulmonary anatomy is challenging, due to the high variability and its three-dimensional (3D) shape. While demands in thoracic oncologic surgery are increasing, the transition from open to thoracoscopic surgery is hampering anatomical understanding. This study analyzed the value of a 3D printed lung model in understanding and teaching anatomy.

**Methods:**

A 3D pulmonary model was created and tested among different levels of proficiency: 10 experienced surgeons, 10 fellow surgeons and 10 junior residents. They were tested in interpretation of anatomy based on thoracic CT-scans, either using the 3D model or a 2D anatomical atlas. Accuracy of the given answers, time to complete the task and the self-reported level of certainty were scored in each group.

**Results:**

In the experienced surgeons group there was no difference in between the 2D-model or 3D-model with a high rate of correct answers in both groups, and no differences in time or certainty. Fellow surgeons highly benefitted from the 3D-model with an improved accuracy from 26.6% to 70.0% (p = 0.001). Time to complete the task was shorter (207 versus 122 s, p < 0.0001) and participants were more secure (median of 4 versus 3, p = 0.007). For junior residents time to complete the task was shorter, the level of certainty was higher, but there was no improvement in accuracy.

**Conclusions:**

3D printing may benefit in understanding anatomical relations in the complex anatomy of the bronchiopulmonary tree, especially for surgeons in training and could benefit in teaching anatomy.

**Supplementary Information:**

The online version contains supplementary material available at 10.1007/s40670-023-01807-x.

## Introduction

**Fig. 1:**
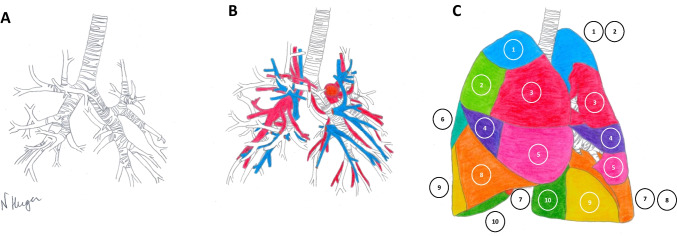
The complexity of the pulmonary anatomy from the right anterolateral side. **A**) The tracheobronchial tree and intrapulmonary airways form the backbone of the pulmonary anatomy. **B**) The pulmonary arteries (red) originate from the right ventricle and the pulmonary veins (blue) drain into the left atrium. They parallel the bronchial structures, when these split into segmental bronchi. **C**) The pulmonary lobes can be subdivided into bronchopulmonary segments which are portions of lung parenchyma supplied by a specific segmental bronchus and its vessels. Segments are numbered and corresponding bronchi, veins and arteries are named according to the bronchopulmonary segments they supply

Pulmonary anatomy is a challenging part in thoracic surgery, due to the high variability and its three-dimensional (3D) shape. The tracheobronchial tree forms the backbone of the pulmonary hilum. The pulmonary arteries and veins originate from and drain into the heart and form the pulmonary circulation. They run along with the subdivision of the bronchus into the intrapulmonary airways (Fig. 1). Thoracic surgeons are taught thoracic anatomy mainly through anatomical text books and by interpreting two-dimensional (2D) imaging. Hands-on training is an essential part in surgical training as it aids in projecting theoretical knowledge to the intra-operative situation. The transition from open to thoracoscopic surgery is hampering this learning curve, due to the limitations in intraoperative tissue mobility and tactile feedback.

At the same time there is a transition from lobar to sublobar resections to treat early-stage lung cancer, localized benign lesions and pulmonary metastases by organ preserving strategies [1]. This development demands improved anatomical knowledge both during preoperative planning and during the surgical procedure [2, 3]. Sublobar anatomy is highly variable and intersegmental divisions are difficult to determine on computed tomography (CT). 3D printing has emerged as a diagnostic tool that may improve visualization, especially in fracture management [4]. In thoracic surgery, the value of 3D printing has been explored limitedly. So far it has been demonstrated to benefit clinicians in their preparation for highly complex procedures [5, 6]. The costs and time investment in preparation of patient specific models are some of the disadvantages in 3D printing in surgery in general. It is therefore important to determine the role of 3D printed models in sublobar surgery and surgical education and clinical practice.

The aim of this study was to identify the possible role of pulmonary 3D printed models for clinical use and the potential of the model in teaching and training purposes. Three groups with varying levels of experience in thoracic surgery compared a 3D printed model with a 2D model and CT scan for either preoperative planning in sublobar surgery or anatomical understanding.

## Materials and Methods

### Participants

Three groups of participants from various training hospitals in the Netherlands were selected based on experience in pulmonary surgery. The ‘experienced surgeons’ consisted of 10 thoracic surgeons who had finished surgical training at least 2 years ago (post-surgical training experience ranged from 2 to 30 years). The ‘fellow surgeons group’ consisted of 10 surgeons who had finished surgical training less than two years ago or were close to finishing their training in thoracic surgery. The ‘junior resident group’ consisted of 10 surgical residents who were not experienced in thoracic surgery. Given the different levels of experience between the groups the expert group tested the clinical value of the model for an advanced surgical technique and the fellow group and junior resident group used the model to answer questions regarding anatomical relationships between different structures of the bronchopulmonary tree. The Institutional Review Board of Rijnstate approved the research protocol.

### Development of the Pulmonary Model

**Fig. 2:**
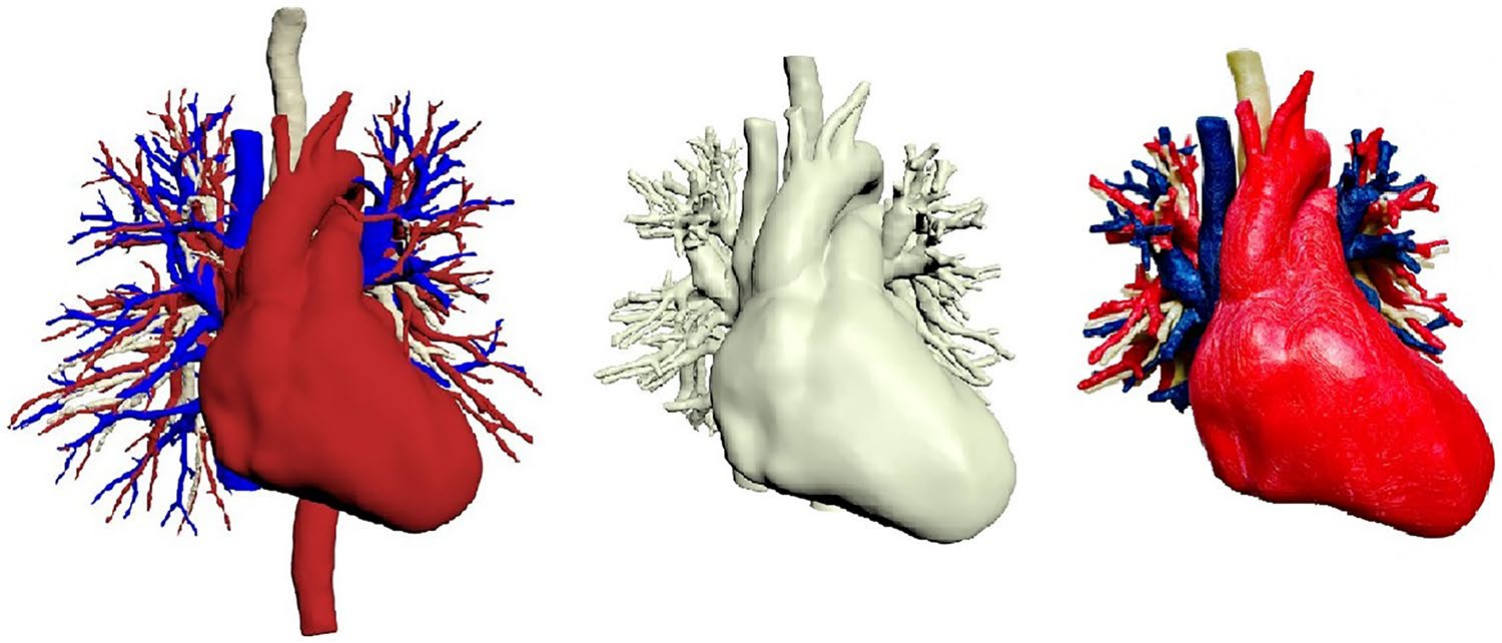
Example of the 3D printed model. From left to right the development of the model from threshold segmentation, to limiting peripheral structures and the 3D printed prototype

Based on CT scan with contrast, the 3D model was created using automatic threshold segmentation of the different anatomical structures of interest. Separate 3D models were created for the pulmonary arteries, pulmonary veins, heart, trachea and bronchi. After reconstruction, the different 3D models were combined and manually smoothed to improve the visual representation and the printability of the model. The structures were cut-off after the third and before the fourth divisions of the bronchi. Finally, notches were created in the visual 3D model for the placement of magnets to indicate different lung segments with separate labels (Fig. 2). The anatomical 3D models were saved as Surface Tessellation Language (STL) data. As a supplementary file to the current publication, the STL file of the 3D model is available for download and can be printed (Supplementary file S1). The authors have licensed the model as CC BY-NC-SA 4.0 (Attribution-NonCommercial-ShareAlike under 4.0 International).

### 3D Printing

Open-source software (Autodesk Meshmixer) was used to prepare the STL data for the 3D printer. The anatomical model was 3D printed in a scale of 1:1 using our in-hospital 3D printer: the Ultimaker S5 (Ultimaker, Utrecht, The Netherlands).

### Segmentectomy

To address both aims of the study, participants were examined according to their level of proficiency. Sixteen CT scans were selected from patients who were eligible to undergo a segmentectomy (< 2 cm nodule, located in the periphery of the lung parenchyma) [7]. The expert surgeons were asked to examine these CT scans and to determine the segment in which the nodule was located. The correct location of the nodule was determined by authors N.H. and E.H. (both thoracic surgeons with experience in segmental resections), independently. The surgeon was asked to use the 2D printed version of the bronchopulmonary segments from the Atlas of Human Anatomy by Netter (7^th^ Edition, 2018, Elsevier) for the first 8 CT scans and used the 3D printed model as an addition for examination of the second 8 CT scans [8]. To avoid a learning effect during the study, half of the surgeons used the 3D model first and the other half used the 2D model first. To avoid bias introduced by complexity of the CT scans, these were allocated to the surgeons in a randomly assigned order. Time to completion of the task was registered. The CT scan on which the 3D model was based was not part of the CT scans that were examined.

### Anatomical Understanding

To determine the role of 3D printing in anatomical understanding of bronchopulmonary anatomy the fellow surgeons and junior residents were asked to answer six anatomical questions regarding a patient case that was presented in a CT scan. The participants were handed an exact 3D model that was based on the patient specific CT scan. For half of the questions the 3D model could be used, for the other half the questions were answered using the CT scan [8]. Questions were randomly allocated to the participants and the use of the 3D or 2D model first was equally distributed among the groups. Time to completion of the tasked was registered.

### Level of Certainty and Overall Impression

Participants from all three groups were asked to rate their level of certainty for the task on a 5-point Likert scale [9]. Herewith the lowest score indicated a low rate of certainty and the highest score indicating high level of certainty regarding the given answer. Moreover, the participants were asked to comment on the model and its potential use. The participants were asked to score on a 5-point Likert scale questions related to the contribution of the 3D model to the given answer, with the lowest score indicating that the 3D model did not contribute at all and the highest score indicating that the 3D model was highly contributive. Finally, the likelihood of using the 3D model in the future was scored on a 5 point Likert scale as well.

### Statistical Analysis

One-way ANOVA was used to assess the difference in time (in seconds) to complete the task between the 3D and 2D model. Pearson’s Chi-Square test was used to compare differences in the frequency distributions of categorical variables between groups. Tests were two-sided and *p* < 0.05 was considered statistically significant. Statistical analyses were performed with statistical software package SPPS 24.0 (Armonk, NY: IBM).

## Results

### Expert Group

**Fig. 3:**
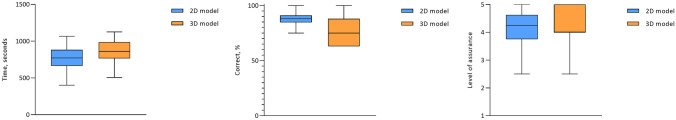
Comparing assessment time, accuracy and level of certainty with the use of a 2D model and 3D model in a group of experienced surgeons

In the experienced surgeons group there was no difference in outcome when either the 2D model or 3D model was used (Fig. 3). The accuracy of the examination was 87.5% when using the 2D model and 77.5% when using the 3D model (p = 0.171). The time to complete the task was on average 89 s shorter with the 2D model, although this was not statistically significantly different between the 2D or 3D model (p = 0.17). Similarly, the level of certainty regarding the selection of the correct segment was not different between both models (p = 0.88).

### Anatomical Understanding

**Fig. 4:**
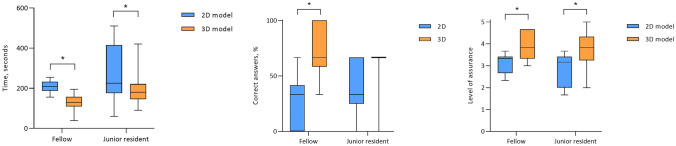
Comparing assessment time, accuracy and level of certainty with the use of a 2D model and 3D model in a group of fellow surgeons and junior residents

The fellow surgeons and junior residents performed the same tasks. In the fellow surgeons group the use of the 3D model led to better achievements in determination of anatomical relations compared with the CT scan, with a higher rate of correct answers (Fig. 4). By using the 3D model the accuracy rate improved from 26.6% to 70.0% (p = 0.001). Also, the time to complete the task was shorter when the 3D model was used (207 versus 122 s, p < 0.0001) and participants were more secure regarding the answer they had given when using the 3D model (median score of 4 versus 3, p = 0.007).

For the junior residents the use of the 3D model did not improve the rate of correct answers significantly, as the use of the 2D model led to 40% correct answers and the 3D model to 60% correct answers (p = 0.121). The time to complete the task was shorter with the use of the 3D model (195 versus 275 s; p = 0.008). There was a higher level of certainty when the 3D model was used to answer the questions (median score of 4 versus 3; p = 0.028).

### Overall Impression

The participants were asked to reflect on the use of the 3D model. The experienced surgeons noticed that the use of a general 3D model could not solve the issues that arise during preoperative planning for segmentectomy. They suggested the use of a patient specific 3D printed model, but only in selected complex cases. Noteworthy, the experienced surgeons with limited experience in segmentectomies were more in favor of the general 3D model than those with more experience. There was a high benevolence towards using the model for training purposes. Both the fellow surgeons and junior residents were in favor of the 3D model, due to the benefit they experienced during the assessment of the CT scan. They ranked high scores on the likelihood to use the model in the future (median score of 4).

## Discussion

The complexity of pulmonary anatomy and increasing use of organ sparing surgery requires an optimal anatomical preparation of the thoracic surgeon. The use of a high-resolution 3D printed anatomical pulmonary model may aid in this purpose. This study demonstrates that the use of such a model is foremost beneficial for training purposes in both fellow surgeons and junior residents.

This study is the first study to assess the role of 3D printing in groups with varying levels of experience. By including 30 participants with a total of 280 measurements substantial data could be derived to analyze the role of 3D printing.

There is ample evidence that medical students find adequate spatial understanding of anatomy based on a 2D image challenging and that 3D visualization improves understanding [10, 11]. This study confirms that surgeons in training benefit from a 3D printed bronchopulmonary model, leading to a reduction in assessment time, an increase in accuracy and a higher level of certainty. These benefits account primarily for participants in the fellow group, which is indicative that a certain level of anatomical knowledge is necessary to benefit from the 3D printed model for correct interpretation. The 3D model supports spatial assessment of the anatomy, giving the opportunity to look at the anatomical structures and their mutual relationship from different angles. This accelerates anatomical comprehension compared with conventional methods, such as 2D printed images. This is in line with a previous meta-analysis on the use of 3D printing in teaching human anatomy [12]. Moreover, it substantiates the use of a 3D model for educational purposes as it has been demonstrated that using 3D printing may even improve training [13].

On the other hand, the role of a non-patient specific model is not beneficial to those who are already experienced in thoracic and pulmonary surgery. Many years of experience in assessment of pulmonary imaging was given as a potential explanation by the experts for this. However, it has been addressed by the experienced surgeons that patient specific 3D models in complex pulmonary anatomy, especially for sublobar surgery or in case of resections beyond anatomical borders could still be of additional value. In sublobar surgery the anatomical variations and understanding of anatomical relations can be rather challenging. It has been emphasized by the thoracic surgical community that preoperative imaging and visualization of anatomical structures is essential in surgical planning.

Notably, the process of 3D printing, especially for the complex and extensively detailed anatomy of the lung unequivocally leads to a time-consuming procedure of data processing and printing. Transferring the data of a DICOM file into a printable model is done manually and the model that was used in the current study took up to 72 h to print, which we believe may hamper broad implementation in clinical practice. It is therefore desirable that a printed model can be used repeatedly, which is the case when it is used for educational purposes. Nevertheless, this study demonstrates that development of an accurate and detailed 3D model based on a patient specific CT scan is feasible. Commercially available anatomical models usually do not give insight into 3D anatomical relations to a full extent, since the bronchopulmonary tree is at least partially embedded within the parenchyma in these models to reduce vulnerability. Moreover, they are not developed using 3D printing technology and concessions are made to the anatomy. Thereby they are not representing the real-life situation, rendering them unsuitable to depict sublobar anatomy. We demonstrate that technical challenges, such as vulnerability of peripheral structures which may hamper 3D printing can be overcome, paving the way for patient specific models.

Improvements in preoperative visualization of anatomical structures are supported by the emerging field of 3D imaging. This involves the conversion of conventional CT scans into 3D animations, and display using virtual reality, augmented reality and holograms [14–17]. Other imaging solutions such as 3D CT may provide a solution for this problem, as the use of 3D CT may be comparable to 3D printing [18]. Additionally, combining virtual reality and artificial intelligence enables 3D visualization of the bronchial anatomy and enables patient specific modeling [17].

In conclusion, 3D printing may benefit in understanding anatomical relations in the complex anatomy of the bronchopulmonary tree, especially for surgeons in training and would benefit in teaching anatomy.

## Supplementary Information

Below is the link to the electronic supplementary material, which contains the STL files of the 3D lung model and the stand on which the 3D model can be rested.

Supplementary file1 (STL 83090 KB)

Supplementary file2 (STL 1425 KB)

## Data Availability

The data that supports the findings from this study is available from the corresponding author upon request.
